# Melatonin Alleviates the Toxicity of High Nicotinamide Concentrations in Oocytes: Potential Interaction with Nicotinamide Methylation Signaling

**DOI:** 10.1155/2021/5573357

**Published:** 2021-04-08

**Authors:** Marwa El-Sheikh, Ahmed Atef Mesalam, Seok-Hwan Song, Jonghyeok Ko, Il-Keun Kong

**Affiliations:** ^1^Division of Applied Life Science (BK21 Four), Gyeongsang National University, Jinju 52828, Republic of Korea; ^2^Department of Microbial Biotechnology, Genetic Engineering and Biotechnology Division, National Research Centre (NRC), Dokki, Cairo 12622, Egypt; ^3^Department of Therapeutic Chemistry, Division of Pharmaceutical and Drug Industries Research, National Research Centre (NRC), Dokki, Cairo 12622, Egypt; ^4^The King Kong Corp. Ltd., Gyeongsang National University, Jinju 52828, Republic of Korea; ^5^Institute of Agriculture and Life Science, Gyeongsang National University, Jinju 52828, Republic of Korea

## Abstract

Despite the numerous studies on melatonin and nicotinamide (NAM, the active form of vitamin B3), the linkage between these two biomolecules in the context of signaling pathways regulating preimplantation embryo development has not yet been investigated. In this study, we used bovine oocyte model to elucidate the effect of melatonin on the developmental competence of oocytes under the stress of high NAM concentrations. Results showed that NAM (20 mM) administration during in vitro maturation (IVM) significantly reduced oocyte maturation and actin distribution, while induced reactive oxygen species (ROS) accumulation and mitochondrial dysfunction, the multiple deleterious effects that were alleviated by melatonin (10^−7^ M). The RT-qPCR and/or immunofluorescence showed upregulation of the apoptosis (Caspase-3, Caspase-9, and BAX), autophagy (Beclin-1, LC3A, LC3B, ATG7, LAMP1, and LAMP2), cell cycle (P21, P27, and P53), and DNA damage (COX2 and 8-OxoG) specific markers in oocytes matured under NAM treatment, compared to NAM-melatonin dual-treated and the untreated ones. In addition, the total cleavage and blastocyst development rate, as well as the total number of cells and the inner cell mass (ICM) per blastocyst, were reduced, while DNA fragmentation was induced, in the group of NAM sole treatment than NAM-melatonin cotreatment and control. Inspecting the underlying mechanisms behind NAM-associated toxicity revealed an increase in transcription pattern of NAM methylation (NNMT and AHCY) genes in NAM-treated oocytes while the opposite profile was observed upon melatonin supplementation. In conclusion, to our knowledge, this is the first study reporting that melatonin can protect oocytes and embryos from NAM-induced injury through its ROS-scavenging activity together with potential interaction with NAM methylation signaling.

## 1. Introduction

The oxidative stress, due to reactive oxygen species (ROS) accumulation, is a key factor that can limit the structural and functional integrity of oocytes, leading to poor developmental competence. Melatonin (N-acetyl-5-methoxytryptamine), a hormone mainly secreted by the pineal gland, has been shown to maintain oocyte quality and embryonic development through the direct protection against oxidative stress [1–3]. It can also trigger the activation of different antioxidant enzymes, such as superoxide dismutase (SOD) and glutathione (GSH) in oocytes [4, 5], and enhance the quality of embryos through upregulating the genes essential for development and cryotolerance [6]. Under heat shock, prolonged culturing, vitrification, low quality of oocytes, the presence of toxic and antidevelopmental compounds, and other stress conditions, melatonin showed beneficial roles in maintaining oocyte maturation and embryo development [4, 7–11].

On the other hand, nicotinamide (NAM), a water soluble form of niacin (vitamin B3), is a dietary supplement that controls several conditions and diseases including cell survival, inflammations, cancer, and metabolic disorders [12]. Supplementation of NAM at low concentrations during in vitro maturation (IVM) significantly improved the developmental competence of oocytes and embryos [13]. However, exposure to high NAM concentrations has shown many adverse effects comprising obesity, liver toxicity, growth inhibition, DNA damage, risk of thrombocytopenia, epigenetic modifications, and cancer progression [14–17]. Moreover, it induced apoptosis, spindle defects, and mitochondrial dysfunction that significantly interfered with the developmental competence of oocytes and embryos, albeit the cellular signaling pathways regulating these adverse effects have not been entirely elucidated [13, 18, 19]. According to the global NAM market report in 2017, the consumption rate of NAM has increased by 30% [17]. Although this might reflect the public awareness of the beneficial role of NAM, the potential adverse effects of high doses of such dietary vitamin should be also considered.

Inside the cells, NAM is generally metabolized to NAM mononucleotide (NMN), by NAM phosphoribosyltransferase (NAMPT), which is converted to NAM adenine dinucleotide (NAD^+^) by NAM mononucleotide adenylyltransferases 1-3 (NMNAT1-3) [20]. The vital role of NAD^+^, generated via NAM-mediated metabolism, in regulating metabolic homeostasis and activating the key enzymes responsible for cellular survival and longevity highlights the therapeutic potential of NAM [21]. However, under excessive NAM accumulation, the methylation pathway modulated by NAM-N-methyltransferase (NNMT) to generate methylated NAM (N-methyl-nicotinamide, metNAM) [22] and the direct oxidation of NAM to form NAM-N-oxide through the effect of cytochrome P450 2E1 (CYP2E1) [23] are two metabolic pathways that can be activated. Unlike the normal conditions, NNMT is induced under the increase in the dietary NAM intake, which accordingly catalyzes the NAM hypermethylation pathway where different underlying metabolites are produced [24]. These include N-methyl-2-pyridone-5-carboxamide (2-PY) and N-methyl-4-pyridone-5-carboxamide (4-PY), via the action of aldehyde oxidase (AOX) [17], and homocysteine (HCY), by the activity of the adenosylhomocysteinase (AHCY) [17, 25].

The reported toxicity of NAM following the synthesis of metNAM can be attributed to the disruption of methionine methylation cycle. The universal methyl donor S-adenosylmethionine (SAMe) level is expected to be consumed by NAM to produce metNAM and S-adenosylhomocysteine (SAH), which is converted to HCY by the activity of AHCY, also known as SAH hydrolase [15, 17, 26]. Hyperhomocysteinemia (HHCY), an elevated level of HCY, is a cytotoxic condition involved in cardiovascular disorders, Alzheimer, Parkinson's, inflammations, and heart diseases [27, 28]. Also, HHCY is associated with reduced fertility, risk of recurrent miscarriage and placental infarction, pregnancy loss at early stages, and premature birth with high incidence of congenital defects [27, 29]. Additionally, elevated levels of follicular HCY were associated with the poor qualities of oocytes and embryos in polycystic ovary syndrome patients undergoing assisted reproduction [30]. The mechanism of the HCY-induced damage is most likely accredited to the direct formation of high ROS levels, inhibition of the antioxidant defense system, and induction of proinflammatory responses, mitochondrial dysfunction, methylation related disorders, and epigenetic defects [15, 28, 31].

Despite the enormous studies on the antioxidant activity of melatonin, no data are available on the potential interplay between NAM and such hormone in the context of oocyte maturation and embryo development. In the current study, we sought to explore the effects and the underlying mechanisms of high NAM concentration on bovine oocytes in the presence and absence of melatonin. To achieve this, NAM and melatonin were administered during IVM, while oocyte maturation, actin-based cytoskeletal complex formation, developmental competence of embryos, ROS levels, mitochondrial distribution, apoptosis, autophagy, and DNA damage were inspected. Additionally, NAM methylation signaling pathway was also scrutinized in oocytes.

## 2. Materials and Methods

### 2.1. Ethical Approval and Chemicals

The experiments were performed according to the guidelines of Gyeongsang National University and under the regulations of the Institutional Animal Care and Use Committee (Approval ID: GAR-110502-X0017). All chemicals were purchased from Sigma-Aldrich (St. Louis, MO, USA) unless otherwise described.

### 2.2. Oocyte Aspiration and In Vitro Maturation (IVM)

The ovaries of the Hanwoo cows were collected at a local slaughterhouse, transported in thermal bottles to the laboratory within 2 h from slaughtering, and washed in sterile physiological saline. Using 18-gauge needles, cumulus-oocyte complexes (COCs) were collected in 50 mL tube containing TL-HEPES (10 mM HEPES, 2 mM sodium bicarbonate, 114 mM sodium chloride, 0.34 mM sodium biphosphate, 10 mM sodium lactate, 0.5 mM magnesium chloride, 2.0 mM calcium chloride, 3.2 mM potassium chloride, 1 *μ*L/mL phenol red, 100 IU/mL penicillin, and 0.1 mg/mL streptomycin). After washing in TL-HEPES, COCs with at least three layers of cumulus cells were picked up under stereomicroscope (Olympus SZ51, Tokyo, Japan) and washed four times in IVM medium (TCM-199 supplemented with 10% (v/v) fetal bovine serum (FBS), 1 *μ*g/mL estradiol-17*β*, 10 ng/mL epidermal growth factor (EGF), 10 *μ*g/mL follicle-stimulating hormone (FSH), 0.2 mM sodium pyruvate, 0.1 mg/mL streptomycin, 0.6 mM cysteine, and 100 IU/mL penicillin). The COCs were distributed into four-well plates containing 700 *μ*L IVM at density of 50 COCs per well in the presence or absence of melatonin and NAM and incubated at 38.5°C and 5% CO_2_ for 22 h. All experiments comprised three groups corresponding to 20 mM NAM, combination of 10^−7^ M melatonin and 20 mM NAM, and the untreated control.

### 2.3. In Vitro Fertilization (IVF)

For fertilization of oocytes, the liquid nitrogen-frozen spermatozoa were thawed and diluted in prewarmed Dulbecco's phosphate-buffered saline (DPBS), then centrifuged at 1800 rpm for 5 min at room temperature. Sperm pellets were resuspended in 500 *μ*L of 20 *μ*g/mL prewarmed heparin supplemented with IVF medium (Tyrode's lactate solution with 22 mg/mL sodium pyruvate, 6 mg/mL bovine serum albumin (BSA), 0.1 mg/mL streptomycin, and 100 IU/mL penicillin) and incubated at 38.5°C and 5% CO_2_ for 15 min. Concentrated sperm was then diluted in IVF medium to a density of 1 − 2 × 10^6^ spermatozoa/mL. Each well of COCs was loaded with 700 *μ*L of prepared sperm followed by incubation at 38.5°C and 5% CO_2_ for 18-20 h.

### 2.4. In Vitro Culture (IVC) and Embryo Development

Following fertilization, cumulus cells were detached by successive pipetting; then, presumed zygotes were maintained in 700 *μ*L complete SOF medium [32] and incubated at 38.5°C and 5% CO_2_. After three days (day 4 postfertilization), total cleavage and the number of 8-16 cell-stage embryos were recorded while the SOF medium was refreshed before incubating the plates for another four days. At day 8 postfertilization, blastocyst development rates were recorded while blastocysts were collected in 4% paraformaldehyde (PFA) and stored at 4°C until use.

### 2.5. Assessment of Oocyte Maturation

Twenty-two hours from the onset of maturation, denuded oocytes, collected after repeated pipetting of COCs, were washed in PBS and the first polar body extrusion was directly visualized under microscope. To determine the stage of maturation, oocytes, incubated with 0.5% Triton X-100 for 20 min, were stained with 4′,6-diamidino-2-phenylindole (DAPI: 1 *μ*g/mL; Thermo Fisher Scientific, Waltham, MA, USA) for 15 min followed by visualization under confocal laser-scanning microscope (Olympus Fluoview FV1000, Tokyo, Japan). Based on the morphology of the nuclear material, the maturation stage of oocyte was classified as follows: germinal vesicle (GV), metaphase of the first meiosis (metaphase I: MI), and metaphase of the second meiosis (MII: mature).

### 2.6. Visualization of Cytoskeleton

The filamentous actin (F-actin) was investigated using Alexa Fluor 488-conjugated phalloidin staining (Thermo Fisher Scientific). In brief, denuded oocytes (*n* = 10‐15: triplicate) were fixed in 4% PFA, washed in PBS, and permeabilized with 0.5% Triton X-100 for 15 min. After washing in PBS, oocytes were incubated with Alexa Fluor 488-conjugated phalloidin for 40 min then washed and stained with DAPI for 15 min at room temperature. Oocytes were visualized under confocal laser-scanning microscope where the fluorescence intensities, in cytoplasm and zona pellucida/oolemma, were estimated using the ImageJ software (National Institutes of Health, Bethesda, MD, USA; https://imagej.nih.gov/ij/).

### 2.7. Measurement of Reactive Oxygen Species (ROS) Levels

Denuded oocytes (*n* = 10‐15: triplicate) were incubated with the ROS indicator 2,7-dichlorodihydrofluorescein diacetate (H_2_DCFDA: 5 *μ*M) for 20 min at 38.5°C followed by three-times washing in PBS and imaging under epifluorescence microscope (Olympus IX71, Tokyo, Japan). Fluorescence intensities of ROS were estimated using the ImageJ software.

### 2.8. Assessment of Mitochondrial Distribution Pattern

To investigate the distribution pattern of mitochondria, denuded oocytes (*n* = 10‐15: triplicate) were incubated with 100 nM MitoTracker deep Red stain (Invitrogen/Molecular Probes, Eugene, OR, USA) at 38.5°C for 40 min before fixation in 4% PFA. Oocytes were inspected under epifluorescence microscope where mitochondrial distribution pattern was classified either as aberrant (dispersed peripherally or semiperipherally in the cytoplasm) or homogeneous (uniformly distributed throughout the cytoplasm).

### 2.9. RNA Extraction and cDNA Synthesis

Total RNA was extracted from oocytes (*n* = 50: triplicate) using Arcturus PicoPure RNA isolation kit (Arcturus, Foster, CA, USA) according to the manufacturer's guidelines. Fixed amounts of RNA (100 ng) were subjected to cDNA synthesis using iScript cDNA synthesis kit (Bio-Rad Laboratories, Hercules, CA, USA) as follows: RNA (15 *μ*L) was mixed with 5x iScript reaction mixture (4 *μ*L) and iScript reverse transcriptase (1 *μ*L) then incubated at 25°C for 5 min, 42°C for 30 min, and 85°C for 5 min.

### 2.10. Quantitative Reverse Transcription PCR (RT-qPCR)

The RT-qPCR was carried out using iQ-SYBR Green Supermix (Bio-Rad Laboratories) according to the manufacturer's instructions. Briefly, 2 *μ*L of forward and reverse primers mix (Table 1), 5 *μ*L of SYBR Green mix, and 1 *μ*L of nuclease-free water were mixed and distributed into hard-shell 96-well skirted PCR plates (Bio-Rad Laboratories) before adding 2 *μ*L of diluted cDNA (150 ng/*μ*L). Using CFX96 instrument (Bio-Rad Laboratories), the qPCR was performed under the following conditions: 95°C for 3 min, 44 cycles of 95°C for 15 s, 58°C for 20 s, and 72°C for 30 s. Each cDNA sample was applied in duplicate (three biological replicates). The mRNA abundance of genes was estimated using GAPDH as a reference gene where the transcription level of each gene in the untreated control was set as 1.

### 2.11. Immunofluorescence

Following IVM, oocytes (*n* = 10‐15: triplicate) were fixed in 4% PFA, washed thrice in PBS, and permeabilized with 0.5% Triton X-100 for 20 min. After blocking in 10% FBS and 3% BSA, prepared in PBS, for 2 h, oocytes were incubated overnight at 4°C with the primary antibodies raised against Caspase-3, Caspase-9, Beclin-1, microtubule-associated protein 1 light chain 3 beta (LC3B), cyclooxygenase 2 (COX2), and 8-oxoguanine (8-OxoG) (Supplementary table 1). Samples were washed in PBS before adding the Alexa Fluor-conjugated secondary antibodies (Supplementary table 1). After 90 min incubation at room temperature, DAPI was added for 15 min then oocytes were spotted on glass sides and investigated under confocal laser scanning microscope. The fluorescence intensities were estimated using the ImageJ software.

### 2.12. Differential Staining of ICM and TE Cells

Day 8 blastocysts, fixed in PFA, were permeabilized with 0.25% Triton X-100 for 20 min, washed three times in washing buffer (0.1% Tween 20 and 0.1% BSA prepared in PBS), and incubated in blocking buffer (5% BSA prepared in PBS) for 1 h at room temperature. Samples were incubated overnight with anti-CDX2 (caudal-related homeobox 2) antibody (BioGenex, Hague, Netherlands) before washing and incubation with Alexa Fluor-568 donkey anti-mouse IgG at room temperature for 1 h. The CDX2 exclusively localizes in the trophectoderm (TE) and is thereby used to distinguish the TE from the inner cell mass (ICM). The nuclei were stained with DAPI for 15 min then the blastocysts were washed and mounted on glass slides and examined under confocal laser-scanning microscope where the total number of cells (DAPI positive), TE (CDX2 positive), and ICM (CDX2 negative) per each blastocyst were recorded.

### 2.13. Terminal Deoxynucleotidyl Transferase dUTP Nick-End Labeling (TUNEL) Assay

The TUNEL assay for detection of DNA fragmentation was performed using In Situ Cell Death Detection kit according to the manufacturer's instructions (Roche Diagnostics, Indianapolis, USA). In brief, PFA-fixed day 8 blastocysts were washed three times in 0.3% polyvinylpyrrolidone (PVP) prepared in PBS and incubated with 0.5% Triton X-100 and 0.1% sodium citrate for 30 min. Samples were treated with fluorescent-conjugated TUNEL solution at 38.5°C for 1 h; then, the nuclei were stained with DAPI for 15 min. Blastocysts were mounted on glass slides and visualized under epifluorescence microscope where the numbers of bright red spots and DAPI-stained nuclei, indicators for DNA fragmentation, and total number of cells, respectively, were counted in each blastocyst.

### 2.14. Statistical Analysis

The statistical analyses were carried out using GraphPad Prism version 6 (San Diego, CA, USA). The comparison between NAM-treated group versus NAM-melatonin cotreated or the untreated group was performed using Student's *t*-test. Results are presented as the mean values ± the standard error of the mean (SEM). The degree of significance was presented as ^∗^, ^∗∗^, ^∗∗∗^, and ^∗∗∗∗^ when the *P* values were below 0.05, 0.01, 0.001, and 0.0001, respectively. All experiments were repeated at least three times.

## 3. Results

### 3.1. Melatonin Reduces NAM-Associated Impairment of Oocyte Maturation and Actin Stabilization

**Figure 1:**
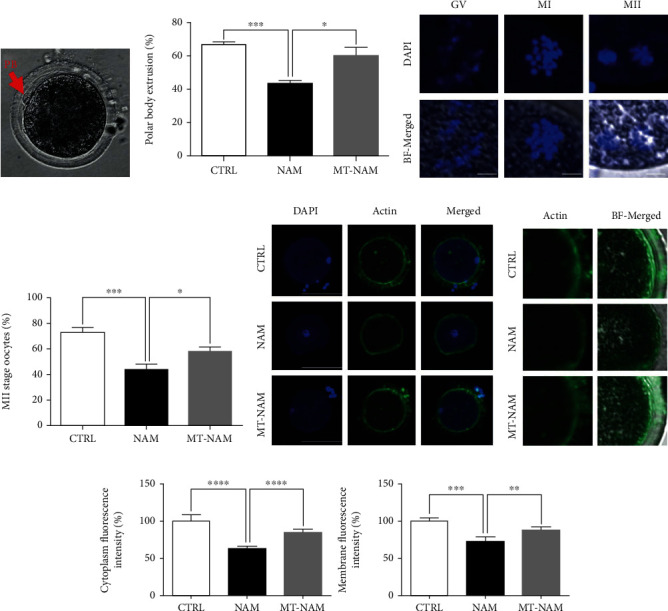
Effect of NAM administration on oocyte maturation in presence and absence of melatonin. (a) Light microscopy for the polar body (red arrow) of in vitro matured oocytes. (b) Percentages of oocytes with extruded polar bodies. (c) Microscopy of nuclear maturation using DAPI staining showing the different stages of maturation. (d) Proportion of oocytes that reached the MII stage. (e, f) Filamentous actin distribution in oocytes using Alexa Fluor 488 phalloidin staining. (g) Fluorescence intensity after phalloidin-based staining in oocyte's cytoplasm and zona pellucida/oolemma. Scale bar = 100 *μ*m. The error bars represent the SEM of measurements of three replicates. PB: polar body; GV: germinal vesicle; MI: metaphase I; MII: metaphase II. BF: bright field; NAM: nicotinamide; MT: melatonin.

We have previously reported that high NAM concentrations can negatively affect the process of embryo development [13]. To investigate whether melatonin can alleviate this effect, bovine oocytes were treated with 20 mM NAM for 22 h in the presence and absence of 10^−7^ M melatonin. The concentration of melatonin was selected based on previous studies on the protective role of melatonin during the IVM of bovine [33, 34], porcine [35], and goat [36] oocytes. Initial inspection of oocyte maturation revealed that NAM at 20 mM concentration significantly reduced polar body extrusion (43.33 ± 1.67%) compared to the untreated control (66.67 ± 1.67%), whereas melatonin supplementation succeeded to restore the normal maturation (60.00 ± 5.00%) (Figures 1(a) and 1(b)). For confirmation, the stage of nuclear maturation was inspected at the end of IVM through DAPI staining of the nuclear material. As shown in Figures 1(c) and 1(d), oocyte maturation was significantly reduced after NAM treatment (44.16 ± 3.70%), compared to the higher levels of melatonin-NAM cotreatment (58.33 ± 3.40%) and control (72.92 ± 3.99%). Representative images for the different stages of oocyte maturation are presented in Figure 1(c).

On the other hand, the effect of NAM and melatonin treatment on the filamentous actin (F-actin) integrity in oocyte's zona pellucida/oolemma and cytoplasm was investigated using phalloidin-based staining. As seen in Figures 1(e)–1(g), the fluorescence intensity of F-actin in oocytes decreased after NAM treatment compared to the untreated control, while significantly increased upon addition of melatonin.

### 3.2. Melatonin Diminishes Oxidative Stress and Mitochondrial Dysfunction in NAM-Treated Oocytes

**Figure 2:**
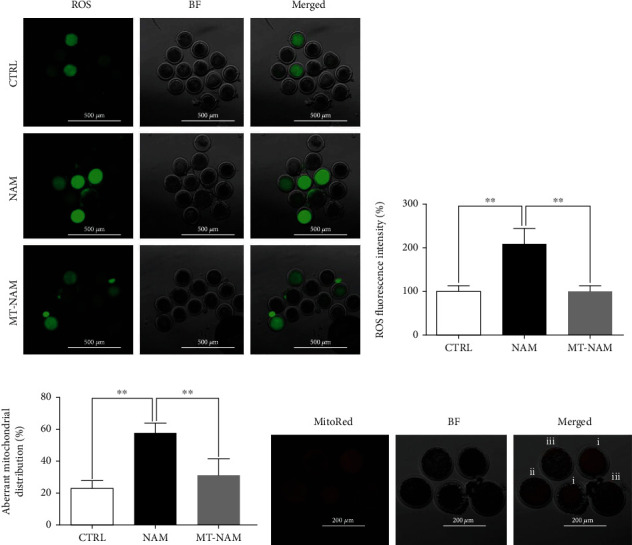
Effect of melatonin and NAM on ROS levels and mitochondrial distribution. (a) Representative images of oocytes stained with 2,7-dichlorodihydrofluorescein diacetate (H2DCFDA). Scale bar = 500 *μ*m. (b) The ROS signals in oocytes. (c) Percentage of oocytes with aberrant (semiperipheral and peripheral) mitochondrial distribution after staining with MitoTracker Red. (d) Representative images of the different mitochondrial distribution patterns showing (i) homogenous, (ii) semiperipheral, and (iii) peripheral distribution. Scale bar = 200 *μ*m. The error bars represent the SEM of measurements of three replicates. ROS: reactive oxygen species; MitoRed: MitoTracker Red; BF: bright field; NAM: nicotinamide; MT: melatonin.

To evaluate the potential of melatonin to scavenge the ROS during NAM treatment, in vitro matured oocytes were coincubated with the ROS-specific stain H_2_DCFDA before visualization under microscope. Remarkably, melatonin supplementation was associated with a significant decrease in the ROS levels compared to the obvious increase under NAM sole treatment (Figures 2(a) and 2(b)). Furthermore, checking the distribution patterns of mitochondria revealed that melatonin can maintain the homogenous mitochondrial distribution, whereas the aberrant distribution was dominant in NAM-treated oocytes compared to melatonin-NAM and control (Figure 2(c)). The morphological appearance of the different mitochondrial distribution patterns is shown in Figure 2(d).

### 3.3. Melatonin Protects Oocytes from NAM-Induced Apoptosis and Autophagy

**Figure 3:**
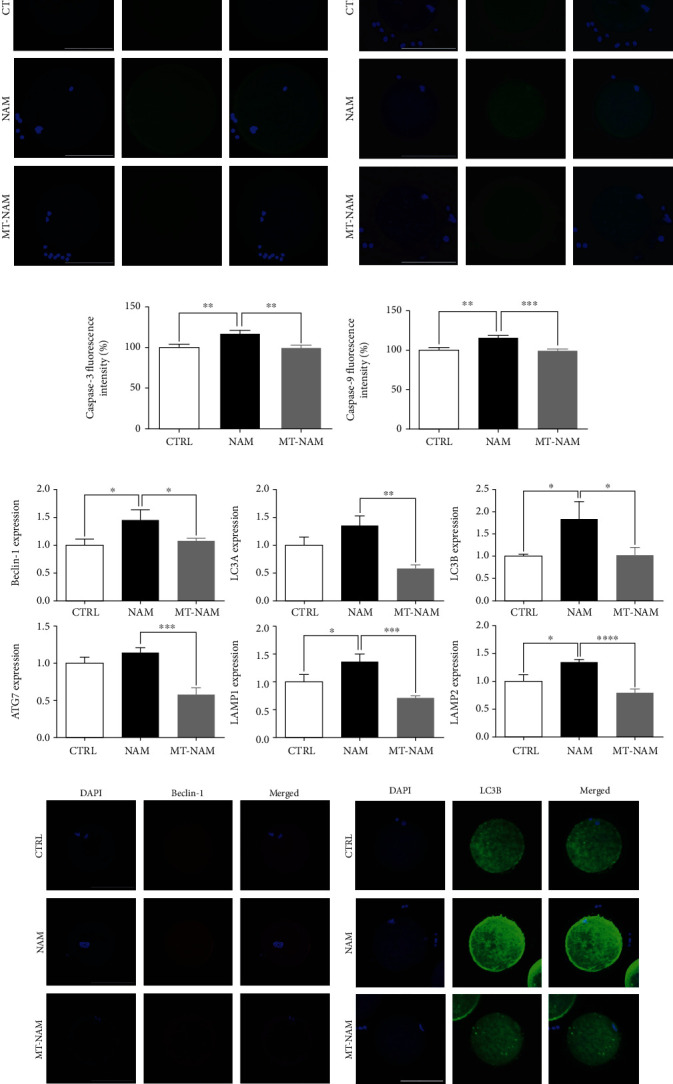
Effect of melatonin/NAM supplementation on apoptosis and autophagy in oocytes. (a) The transcription levels of apoptosis-related genes in matured oocytes. (b, c) Representative images of Caspase-3 and Caspase-9 immunofluorescence. (d, e) Fluorescence intensity of Caspase-3 and Caspase-9 in oocytes. (f) Transcriptional pattern of the different autophagy-related markers in oocytes. (g, h) Representative images of Beclin-1 and LC3B staining. (i, j) Fluorescence intensity of Beclin-1 and LC3B in oocytes. Scale bar = 100 *μ*m. The error bars represent the SEM of measurements of three replicates. BCL2: B-cell lymphoma 2; BAX: Bcl-2-associated X apoptosis regulator; Beclin-1: autophagy-related gene 6; LC3A (MAP1LC3A): microtubule-associated protein 1 light chain 3 alpha; LC3B (MAP1LC3B): microtubule-associated protein 1 light chain 3 beta; ATG7: autophagy-related gene 6; LAMP1: lysosomal-associated membrane protein 1; LAMP2: lysosomal-associated membrane protein 2; NAM: nicotinamide; MT: melatonin.

Moving forward, the effect of NAM and melatonin on the expression level of different apoptosis and autophagy markers was investigated at transcriptional and/or translation levels. As shown in Figure 3(a), upregulation of Caspase-3 and BAX and downregulation of BCL2 were observed in NAM-treated oocytes as compared to control. Contrarily, the addition of melatonin was accompanied with downregulation of Caspase-3 and BAX. Despite the increase in BCL2 level under melatonin-NAM treatment, this effect did not reach the statistical significance (*P* > 0.05). For confirmation, inspecting the protein levels of Caspase-3 and Caspase-9 in oocytes using immunofluorescence showed a significant increase in the levels of these two proteins in NAM-treated oocytes compared to melatonin-NAM cotreatment and control ones (Figures 3(b)–3(e)). Similarly, the transcription patterns of the autophagy-related genes Beclin-1, LC3A, LC3B, ATG7, LAMP1, and LAMP2 and the translation patterns of Beclin-1 and LC3B were investigated in oocytes treated with NAM and melatonin-NAM. As shown in Figures 3(f)–3(j), an induction in these autophagy markers was observed in NAM-treated group compared to melatonin-NAM cotreatment and control.

### 3.4. Melatonin Attenuates DNA Damage and Cell Cycle Arrest in NAM-Treated Oocytes

**Figure 4:**
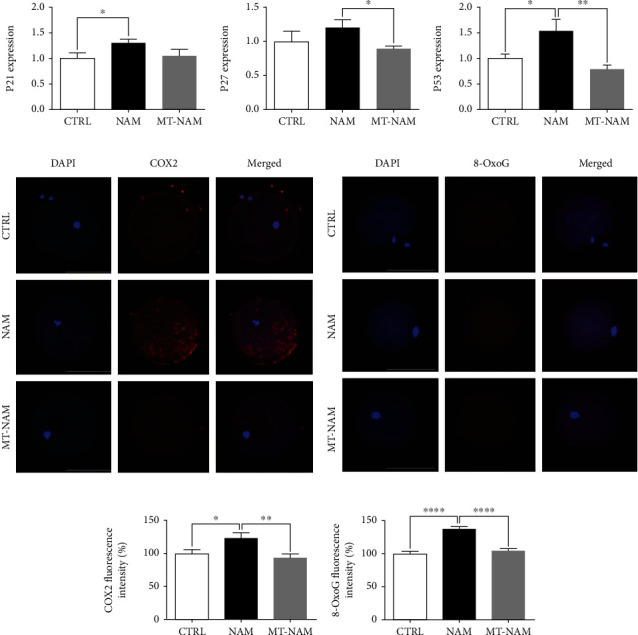
Effect of melatonin and NAM administration on cell cycle regulation and DNA damage in oocytes. (a) Relative expression of markers involved in cell cycle arrest and DNA damage. (b, c) Immunofluorescence of matured oocytes showing COX2 and 8-OxoG expression. (d, e) The fluorescence intensity of COX2 and 8-OxoG. The error bars represent the SEM of measurements of three replicates. Scale bar = 100 *μ*m. P21: cyclin-dependent kinase inhibitor 1A; P27: cyclin-dependent kinase inhibitor 1B; COX2: cyclooxygenase 2; 8-OxoG: 8-oxoguanine; NAM: nicotinamide; MT: melatonin.

To explore whether NAM accumulation can induce a disturbance in cell cycle and DNA repair mechanisms in oocytes, the abundance of the specific markers P21, P27, P53, COX2, and 8-OxoG was investigated using RT-qPCR and immunofluorescence. As seen in Figure 4(a), NAM treatment was associated with upregulation of the mRNA of P21, P27, and P53 compared to melatonin-NAM cotreatment or the untreated control. Protein expression showed an obvious overexpression of COX2 and 8-OxoG under NAM sole treatment whereas the opposite pattern was observed following melatonin administration (Figures 4(b)–4(e)).

### 3.5. Melatonin Restores the Developmental Competence of Embryos Post-NAM Treatment

**Figure 5:**
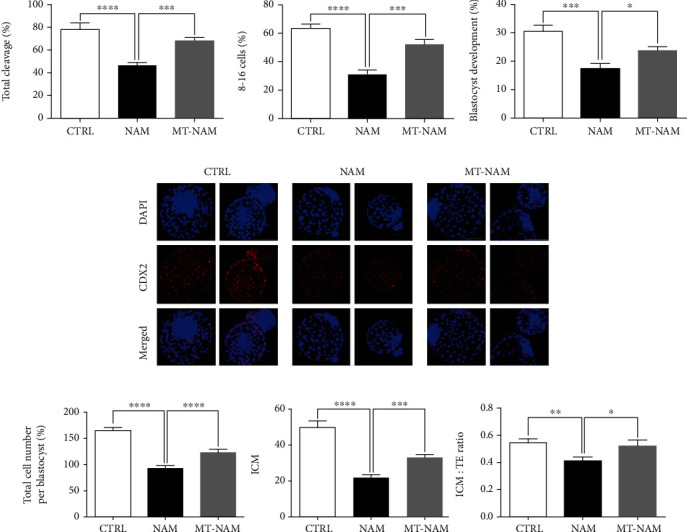
Impact of melatonin and NAM on the developmental competence and quality of embryos. (a) Total cleavage, (b) 8-16 cell embryos, and (c) blastocyst development rates after melatonin/NAM treatment. (d) Representative images of the differential staining using the TE-specific marker CDX2. (e) The total number of cells, ICM cells, and the ICM : TE ratio per blastocyst. Scale bar = 100 *μ*m. The error bars represent the SEM of measurements of at least three replicates. CDX2: caudal-related homeobox 2; TE: trophectoderm; ICM: inner cell mass; NAM: nicotinamide; MT: melatonin.

We went further to check the developmental competence of embryos after NAM treatment in the presence and absence of melatonin. As shown in Figures 5(a) and 5(b), the total cleavage and the number of 8-16 embryos, recorded at day 4 postfertilization, displayed a significant decline under NAM sole treatment (45.83 ± 3.29% for total cleavage and 30.63 ± 3.60% for 8-16 cell stage embryos) compared to the untreated control (77.50 ± 2.54% for total cleavage and 63.07 ± 3.20% for 8-16 cell stage embryos). Interestingly, administration of melatonin succeeded to restore the normal development rates (67.67 ± 2.77% for total cleavage and 52.10 ± 3.25% for 8-16 cell stage embryos). Similarly, day 8 blastocyst development showed the lowest rate in the group of NAM treatment compared to the melatonin-NAM dual-treated and the control groups (17.43 ± 1.72%, 23.57 ± 1.49%, and 30.43 ± 2.13% corresponding to NAM, melatonin-NAM, and control, respectively, Figure 5(c)).

We proceed to investigate the effect of NAM and melatonin treatment on the quality of embryos using differential staining. As shown in Figures 5(d) and 5(e), the total number of cells per blastocyst was significantly lower in NAM-treated group compared to melatonin-NAM derived and the control embryos (92.32 ± 5%, 123.1 ± 6%, and 164.5 ± 6% for NAM, melatonin-NAM, and control, respectively). Since the ICM : TE ratio can be used for evaluating the quality of embryos [37], we used the TE-specific CDX2 transcription factor immunofluorescence to assess the differential staining of blastocysts. Obviously, NAM-treated group exhibited the lowest score of ICM (CDX2-negative) cells and accordingly the ICM : TE ratio compared to control, whereas melatonin succeeded to partially normalize these parameters (Figures 5(d) and 5(e)).

**Figure 6:**
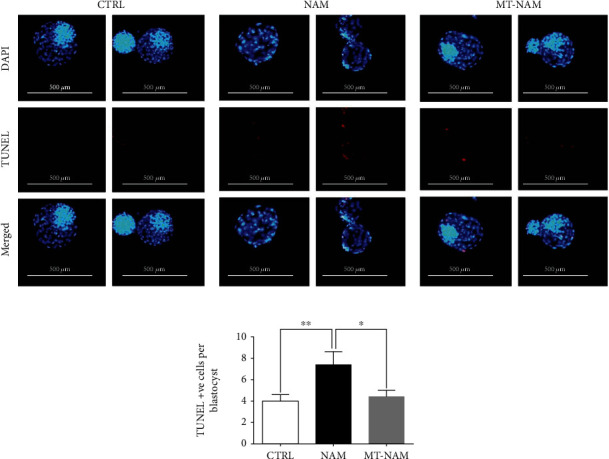
DNA fragmentation analysis after NAM treatment in the presence and absence of melatonin. (a) Representative images for DAPI and TUNEL of day 8 blastocysts. (b) The number of TUNEL positive cells per each blastocyst. Scale bar = 500 *μ*m. The error bars represent the SEM of measurements of three replicates. DAPI: 4′,6-diamidino-2-phenylindole; TUNEL: terminal deoxynucleotidyl transferase dUTP nick-end labeling; NAM: nicotinamide; MT: melatonin.

The potential protective role of melatonin on NAM developed embryos was also inspected through checking the DNA fragmentation using TUNEL assay. The results showed that NAM administration significantly increased, whereas melatonin supplementation succeeded to decrease the number of apoptotic cells in day 8 blastocysts (7.41 ± 1.25%, 4.41 ± 0.59%, and 3.94 ± 0.63% for NAM, melatonin-NAM, and control blastocysts, respectively, Figures 6(a) and 6(b)).

### 3.6. Melatonin Interacts with NAM Methylation Signaling Pathway

**Figure 7:**
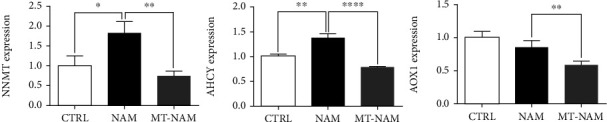
Effect of NAM and melatonin on the transcription patterns of genes involved in NAM methylation signaling in oocytes. The error bars represent the SEM of measurements of three replicates. NNMT: nicotinamide-N-methyltransferase; AHCY: adenosylhomocysteinase; AOX1: aldehyde oxidase. NAM: nicotinamide; MT: melatonin.

To clarify the underlying mechanisms behind the protective role of melatonin against NAM toxicity in oocytes, the transcription patterns of the key genes of NAM methylation signaling pathway were investigated using RT-qPCR. These genes included nicotinamide-N-methyltransferase (NNMT, for production of metNAM), adenosylhomocysteinase (AHCY, for homocysteine (HCY)), and aldehyde oxidase (AOX1, for 2-PY and 4-PY metabolites). As shown in Figure 7, upregulation of the tested genes, except AOX1, was observed in oocytes of NAM sole treatment compared to the untreated control. In addition, the administration of melatonin remarkably downregulated the transcription levels of the three tested genes (Figure 7).

## 4. Discussion

The antioxidant activity of melatonin during the process of embryonic development has been comprehensively studied, whereas the impact of high NAM concentrations on such developmental process has been fewly reported [6, 7, 13, 18, 38]. To date, the potential linkage between melatonin and NAM in the context of embryo development has not yet been clarified. Using the in vitro bovine oocyte model, we sought to elucidate the interplay between these two biomolecules through studying the effect of NAM administration on the different steps of embryo development, in the presence and absence of melatonin.

Oocyte maturation, the progression from GV stage to MII, is an essential step for successful fertilization and embryo development [39]. The distribution of actin was reported to influence oocyte maturation [40, 41]. In the current study, microscopic examination of oocytes showed that NAM at 20 mM was associated with a significant decline in the number of oocytes with obviously extruded polar body and those reached the MII stage, the mature oocytes. It also affected the distribution of filamentous actin in oocytes. Nonetheless, melatonin supplementation under the stress of NAM significantly restored the normal maturation and the actin formation. In line with this, Zhang et al. reported a delay of oocyte maturation and actin distribution in porcine oocytes following exposure to 5 mM NAM [18]. In addition, the ability of melatonin to protect oocytes against the deleterious effects of antidevelopmental compounds including aflatoxin B1, paraquat, and rotenone has been reported [2, 5, 34, 42]. This confirms our observation and reflects the beneficial role of such pineal hormone on oocyte maturation even under the stress of high NAM concentrations.

We have recently reported that exposing oocytes to high dosages of NAM activates the successive release of reactive oxygen species (ROS), which can considerably limit the processes of oocyte maturation and embryo development [13]. In the current study, we moved forward to investigate the potential ROS-scavenging activity of melatonin under the stress of NAM. Interestingly, oocytes matured following melatonin-NAM cotreatment displayed lower ROS levels compared to NAM sole treatment, assuring the, previously reported, protective role of melatonin against different toxic compounds [2, 5, 34, 42].

Overproduction of ROS can induce damage of mitochondria, the cellular organelles responsible for energy production, via ROS-induced ROS release mechanism [43]. Mitochondrial dysfunction has been observed in obesity, diabetes, tumors, and cardiovascular and cerebrovascular diseases [28, 31, 44]. In the current study, higher incidence of aberrant mitochondrial distribution pattern was observed in NAM-treated oocytes. Notably, the majority of oocytes matured under melatonin-NAM supplementation displayed homogenous distribution, an indicator for the quality and developmental competence of oocyte [45]. Consistent with these observations, melatonin enhanced mitochondrial biogenesis that protected the early porcine embryos against mitochondrial damage following rotenone treatment [42]. Altogether, this supports the aforementioned results of maturation and ROS of oocytes matured under melatonin/NAM treatment.

It has been shown that induction of apoptosis and autophagy during IVM negatively affects oocyte maturation and subsequent embryo development [46, 47]. We have previously found an association between high NAM concentrations and the induction of apoptosis and autophagy in oocytes [13]. Considering this, we herein investigated whether melatonin can protect oocyte from apoptosis and autophagy during NAM treatment. Testing the mRNA and/or protein levels of the apoptosis (Caspase-3, Caspase-9, BCL2, and BAX) and autophagy (Beclin-1, LC3A, LC3B, ATG7, LAMP1, and LAMP2) markers showed downregulation of BCL2 and upregulation of the other genes, in oocytes treated with NAM, whereas their levels were normalized by melatonin. The linkage between DNA damage and ROS production, apoptosis, mitochondrial dysfunction, reduced oocyte maturation, cell cycle arrest, and, in some cases, fertility loss has been reported [48, 49]. In the present study, upregulation of DNA damage and cell cycle arrest-related markers P21, P27, P53, COX2, and 8-oxoG was observed in oocytes exposed to NAM, but melatonin significantly restored their normal levels. This corroborates the previous studies that showed higher incidence of ROS production and DNA damage in developing rats after excessive NAM supplementation [15], as well as in oocytes treated with fipronil, an insecticide [48], and confirms the protective role of melatonin against NAM-induced oxidative stress in oocytes.

We went further to investigate whether melatonin administration during IVM can confer a long-term protection in developing embryos. In spite of the toxicity of sole NAM treatment, melatonin succeeded to restore the normal cleavage, 8-16 cell stage embryos, and blastocyst development rates. In addition, higher incidence of DNA fragmentation, lower number of cells per blastocyst, and lower index of ICM : TE were observed in embryos developed from NAM-treated oocytes compared to melatonin-NAM cotreatment or the untreated control. Since the DNA fragmentation and the CDX2-based differential staining are generally used, together with cleavage and blastocyst development rates assessment, for evaluating the quality of preimplantation embryos [50], our observations evidently revealed a protective role of melatonin on developing embryos by improving their quality.

Likewise, we sought to decipher the potential mechanisms behind melatonin and NAM interplay in oocyte. The NAM methylation, mediated by NAM-N-methyltransferase (NNMT), is a principal pathway for NAM metabolism in mammals. The first product of NAM methylation is the N-methyl-nicotinamide (metNAM) which can be further processed to the toxic metabolites 2-PY and 4-PY via the activity of aldehyde oxidase (AOX) [22–24]. In the current study, checking the expression pattern of the key genes involved in NAM methylation showed a dramatic increase in the transcription level of NNMT under the stress of NAM, the effect that was strongly abrogated by melatonin. Although the AOX1 level in oocytes treated with NAM did not show a significant difference compared to control, it was significantly downregulated upon melatonin supplementation. In line with our results, Kang-Lee et al. reported that NAM administration in rats was associated with an increase in metNAM and not 2-PY and 4-PY levels [24].

To clarify the possible involvement of other alternative metabolic pathways for metNAM rather than the AOX1 upregulation, the transcription pattern of the adenosylhomocysteinase (AHCY), the enzyme that converts S-adenosylhomocysteine (SAH) to homocysteine (HCY) within the S-adenosylmethionine (SAMe) methylation cycle, was investigated. The NNMT utilizes SAMe to produce metNAM and SAH, converted to HCY by AHCY [17]. Interestingly, a dramatic increase in the expression level of AHCY was observed in NAM-treated oocytes whereas a strong downregulation was witnessed after addition of melatonin. In line with our findings, the involvement of HCY in the induction of mitochondrial dysfunction in rat ischemic brain [31] and reproduction disorders including reduced fertility, suppressed fertilization, and the developmental competence of oocytes and embryos, increased risk of recurrent miscarriage, placental infarction, and congenital defects was reported [27, 29, 30]. In addition, the protective role of melatonin against HCY-induced oxidative injury, through decreasing the levels of Caspase-3 and BAX and increasing BCL2, has been observed in human umbilical vein endothelial cells (HUVECs) and hippocampus of rats [51, 52]. Moreover, the high HCY levels observed in pinealectomized mouse highlights a potential linkage between the pineal hormone melatonin and the maintenance of the HCY levels [53].

**Figure 8:**
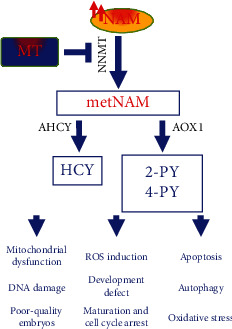
Schematic representation for the protective role of melatonin against NAM-induced toxicity in oocytes. NNMT: nicotinamide-N-methyltransferase; metNAM: N-methyl-nicotinamide; AHCY: adenosylhomocysteinase; AOX1: aldehyde oxidase; HCY: homocysteine; 2-PY: N-methyl-2-pyridone-5-carboxamide; 4-PY: N-methyl-4-pyridone-5-carboxamide; ROS: reactive oxygen species; NAM: nicotinamide; MT: melatonin.

In conclusion, to the best of our knowledge, this is the first study reporting that administration of melatonin during IVM can protect bovine oocytes against high NAM concentration-induced ROS accumulation, apoptosis, DNA damage, mitochondrial dysfunction, and reduced developmental competence of embryos. This can be attributed to a potential involvement of melatonin in regulating NAM hypermethylation signaling and hence alleviating the NNMT- and HCY-induced oxidative stress and mitochondrial dysfunction (Figure 8).

## Figures and Tables

**Table 1:** The primers used in RT-qPCR analysis.

Gene name	Gene sequence	Accession number	Product size
Apoptosis-related genes			
Caspase-3	F: CCCAAGTGTGACCACTGAAC R: CCATTAGGCCACACTCACTG	NM_001077840.1	169
BCL2	F: TGGATGACCGAGTACCTGAA R: CAGCCAGGAGAAATCAAACA	NM_001166486.1	120
BAX	F: CACCAAGAAGCTGAGCGAGTGT R: TCGGAAAAAGACCTCTCGGGGA	XM_027515208.1	118
Autophagy-related genes			
Beclin-1	F: AGTTGAGAAAGGCGAGACAC R: GATGGAATAGGAACCACCAC	NM_001033627.2	100
MAP1LC3A (LC3A)	F: CATGAGCGAGTTGGTCAAAA R: GGGAGGCGTAGACCATGTAG	XM_027558753.1	170
MAP1LC3B (LC3B)	F: TTATCCGAGAGCAGCATCC R: AGGCTTGATTAGCATTGAGC	NM_001001169.1	170
ATG7	F: ATGGCCTTTGAGGAACCTTT R: ATGCCTCCCTTCTGGTTCTT	XM_010817935.3	210
LAMP1	F: GTGAAGAATGGCAACGGAC R: GCATCAGCTGGACCTCGTAA	XM_027558031.1	250
LAMP2	F: AAGAGCAGACCGTTTCCGTG R: CGAACACTCTTGGGCAGTAG	XM_027535042.1	110
Cell cycle and DNA damage-related genes			
P21	F: GCAAATATGGGTCTGGGAGA R: AAATAGTCCAGGCCAGGATG	NM_001098958.2	112
P27	F: TGTCAAACGTGCGAGTGTCTA R: CTCTGCAGTGCTTCTCCAAGT	XM_019961532.1	150
P53	F: CTATGAGATGTTCCGAGAGC R: CTCTCTCTTGAGCATTGGTT	NM_174201.2	153
NAM methylation-related genes			
NNMT	F: CCCAGGTGCTCAAGTGTGAT R: CAGCCTCAAGACACAGGGAG	XM_015474625.2	99
AHCY	F: GCAACTGCTCACTCAGTCCT R: AGGCCTGGATGGTAAAGTGC	NM_001034315.1	81
AOX1	F: AATGTGACCCGGAAACTCCC R: ATGTGGCCCCCTAAAGAAGC	XM_024999354.1	116
Reference gene			
GADPH	F: CCCAGAATATCATCCCTGCT R: CTGCTTCACCACCTTCTTGA	NM_001034034.2	185

## Data Availability

The data that support the findings of this study are available from the corresponding author upon reasonable request.

## Supplementary Materials

Supplementary Materials Supplementary Table 1: antibodies used in immunofluorescence.
