# Supplementary material for: Melatonin Alleviates the Toxicity of High Nicotinamide Concentrations in Oocytes: Potential Interaction with Nicotinamide Methylation Signaling
Source: Oxid Med Cell Longev. 2021 Apr 8;2021:5573357. doi: 10.1155/2021/5573357 (PMC8049830; doi:10.1155/2021/5573357)
Supplement: Supplementary Materials — Supplementary Table 1: antibodies used in immunofluorescence. [file 5573357.f1.docx]

**Supplemental Table S1.** Antibodies used in immunofluorescence.

| **Antibody name** | **Manufacturer** | **Catalog number** | **Dilution** |
| --- | --- | --- | --- |
| **Primary Antibody** | | | |
| Anti-Caspase-3 | Santa Cruz | Sc-1225 | 1:100 |
| Anti-Caspase-9 | Santa Cruz | Sc-8355 | 1:100 |
| Anti-Beclin-1 | Santa Cruz | Sc-48341 | 1:200 |
| Anti-LC3B | Abcam | Ab51520 | 1:1000 |
| Anti-COX2 | Santa Cruz | Sc-376861 | 1:100 |
| Anti-8oxoG | Santa Cruz | Sc-130914 | 1:100 |
| Anti-CDX2 | Biogenex | AM392 | Ready to use |
| **Secondary Antibody** | | | |
| Alexa Fluor-568 donkey anti-mouse IgG | Invitrogen | A10037 | 1:500 |
| Alexa Fluor-488 donkey anti-rabbit IgG | Thermo Fisher | A21206 | 1:500 |
| Alexa Fluor-488 donkey anti-goat IgG | Invitrogen | A11055 | 1:500 |
